# Walking while Performing Working Memory Tasks Changes the Prefrontal Cortex Hemodynamic Activations and Gait Kinematics

**DOI:** 10.3389/fnbeh.2016.00092

**Published:** 2016-05-18

**Authors:** Ming-I B. Lin, Kuan-Hung Lin

**Affiliations:** Department of Industrial and Information Management, National Cheng Kung UniversityTainan, Taiwan

**Keywords:** dual task, NIRS, kinematic, cognitive, gait, obstacle, *n*-back

## Abstract

**Background:** Increasing evidence suggests that walking while performing a concurrent task negatively influences gait performance. However, it remains unclear how higher-level cognitive processes and coordination of limb movements are altered in challenging walking environments. This study investigated the influence of cognitive task complexity and walking road condition on the neutral correlates of executive function and postural control in dual-task walking.

**Methods:** Twenty-four healthy young adults completed a series of overground walks with three walking road conditions (wide, narrow, with obstacles) with and without the concurrent *n*-back working memory tasks of two complexity levels (1-back and 3-back). Prefrontal brain activation was assessed by functional near-infrared spectroscopy. A three-dimensional motion analysis system was used simultaneously to measure gait performance and lower-extremity kinematics. Repeated measures analysis of variance were performed to examine the differences between the conditions.

**Results:** In comparison with standing still, participants showed lower *n*-back task accuracy while walking, with the worst performance from the road with obstacles. Spatiotemporal gait parameters, lower-extremity joint movements, and the relative changes in oxygenated hemoglobin (HbO) concentration levels were all significantly different across the task complexity and walking path conditions. While dual-tasking participants were found to flex their hips and knees less, leading to a slower gait speed, longer stride time, shorter step length, and greater gait variability than during normal walking. For narrow-road walking, smaller ankle dorsiflexion and larger hip flexion were observed, along with a reduced gait speed. Obstacle negotiation was mainly characterized by increased gait variability than other conditions. HbO levels appeared to be lower during dual-task walking than normal walking. Compared to wide and obstacle conditions, walking on the narrow road was found to elicit a smaller decrement in HbO levels.

**Conclusion:** The current study provided direct evidence that, in young adults, neural correlates of executive function and dynamic postural control tend to be altered in response to the cognitive load imposed by the walking environment and the concurrent task during ambulation. A shift of brain activation patterns between functionally connected networks may occur when facing challenging cognitive–motor interaction.

## Introduction

In everyday life, it is common for individuals to be walking while concurrently engaging in another activities, such as passing through a circuitous obstacle course, watching street signs, recalling a shopping list, attending to verbal communication, or interacting with mobile devices. Research has found that pedestrians distracted by mobile/smartphone use tended to show reduced situational awareness, develop inattentional blindness, and increase their unsafe behaviors while crossing the street (Nasar et al., 2008; Hyman et al., 2009; Byington and Schwebel, 2013). Moreover, a decrease in arm swinging has also been reported under the texting and walking condition and can potentially reduce walking stability (Schabrun et al., 2014). Consequently, there is an increased risk of falling, tripping, and other accidental injuries related to phone usage in pedestrians (Byington and Schwebel, 2013; Nasar and Troyer, 2013).

Growing research has adopted the DT walking paradigm to investigate the impact of concurrent secondary tasks on the interplay between gait and cognition (Al-Yahya et al., 2011). Potential DTC may occur when the total demands of attention and execution function required by simultaneously performing two tasks exceed the individual’s information processing capacity, thus leading to negative effects on the performance of either task. Decreased gait speed in response to the additional demands of postural control under dual-tasking has been widely reported in prior studies involving mental arithmetic (Mirelman et al., 2014), VFT (Yogev-Seligmann et al., 2010), and texting on a mobile phone (Schabrun et al., 2014; Plummer et al., 2015). On the other hand, the findings regarding the modification in gait variability in healthy young adults were less consistent and seem more profound in texting while walking (Agostini et al., 2015), but relatively subtle in either sensorimotor tasks (Beurskens and Bock, 2013) or simple mental subtraction tasks (Springer et al., 2006; Mirelman et al., 2014; Francis et al., 2015). Therefore, the observed gait changes could be task dependent, with challenging secondary tasks eliciting greater DT decrements.

Another aspect of locomotion that has received little attention in recent DT studies is the role of walking road conditions. Past research suggests that manipulating foot placement using narrow-based walking or obstacle negotiation can be useful for assessing stability (Schrager et al., 2008; Francis et al., 2015). Unfortunately, only a few studies in the literature have examined the influence of a challenging physical environment while dual-tasking (Kelly et al., 2008; Beurskens and Bock, 2013). Moreover, the non-gait tasks used in the previous two works are relatively easy for healthy young adults and therefore may not completely reflect the rising demand on distinct aspects of executive function experienced by pedestrians distracted by current mobile games. Besides, to the best of our knowledge, we are unaware of research directly quantifying gait adaptation in connection with lower-extremity joint kinematics during DT walking under road conditions with obstacles. Valuable insights could be obtained if we learn how the central nervous system adapts through distinct strategies to achieve the desired postural control while performing a task concurrent with ambulation (Dingwell and Marin, 2006; Perry and Burnfield, 2010).

Technological limitations make it difficult to use fMRI to evaluate the underlying neutral processes associated with gaits during movement. Substituting motor imagery for actual movements, the latest review work has indicated that researchers have used fMRI to investigate brain activity during various locomotion situations in healthy young and old individuals, as well as among neurological patients and blind people (Hamacher et al., 2015), even though caution should be exercised when participants have not physically executed the action to be imagined at a reasonable level (Olsson and Nyberg, 2010). Therefore, in this study, an alternative approach, fNIRS, was chosen to measure the hemodynamic response to cortical brain activation during real walking through neurovascular coupling (Scholkmann et al., 2014; Hamacher et al., 2015). Recently, several studies utilized fNIRS to investigate brain activity during DT walking (Holtzer et al., 2011; Doi et al., 2013; Beurskens et al., 2014; Mirelman et al., 2014). Compared to NW, higher activation in the PFC) was found under the DT condition, i.e., walking with a concurrent verbal alphabet recall task (Holtzer et al., 2011). Moreover, the increase was also significantly higher in the young participants than in their older counterparts. Similarly, the HbO level in the FPC was increased gradually from NW, to walking while counting forward, to walking while serially subtracting 7 s (Mirelman et al., 2014). On the other hand, prefrontal activation in healthy elderly participants was found to decrease during walking with a visual demanding task compared to during NW, whereas few changes were observed in young individuals from single- to DT walking (Beurskens et al., 2014). Moreover, it remains unclear whether the additional demand of executive function imposed by adaptive walking tasks will cause distinct activation patterns in FPC or not.

Thus, the objective of this study was to comprehensively examine the effects of DT interference on the coordination of lower-body movements and brain neural processes in healthy young adults under various cognitive-motor demands. Laboratory-based experiments were designed to allow simultaneous measurement of the behavioral outcomes of a WM task with visual requirements, hip/knee/ankle kinematics, spatiotemporal gait parameters, and the hemodynamic activation of the PFC in single- and DT overground walking. We hypothesized that dual-tasking will increase the demands of executive control and therefore affect lower-limb coordination at both the behavioral and neural levels. Moreover, we also expected the influence of dual-tasking to be more profound in concert with the increase in task demand imposed through various combinations of task complexity and walking road conditions.

## Materials and Methods

### Participants

Twenty-four healthy young adults (12 males, 12 females, 20–27 years old) were recruited from local universities to join the study. All participants had normal or corrected-to-normal vision and were right-handed, as evaluated by the Edinburgh handiness inventory (Oldfield, 1971). None of them had a diagnosis of neurological disease, musculoskeletal disorder, or color-blindness or had experienced medical illness or injuries limiting the ability to walk or text on a smartphone over the past 6 months. A summary of the participants’ anthropometric characteristics and usage experiences of smart phone is presented in **Table 1**. Male participants were generally older, taller, heavier, and had slightly longer hand and thumb length than female counterparts. Both male and female participants, on average, had owned and used touch-screen based smartphones for more than 36 months, with no gender differences observed. The experimental protocols were approved by the Human Research Ethics Committee of the National Cheng Kung University (#103–335). Before participation, written informed consent in accordance with the Declaration of Helsinki was obtained from all individuals included in this study.

**Table 1:** Participant demographic characteristics and smartphone experience.

Variables	Male (*n* = 12)	Female (*n* = 12)	*P*-value
Age (years)	24.0 (±1.3)	22.7 (±1.2)	0.013
Height (cm)^∗^	171.2 (±5.7)	162.1 (±4.8)	<0.001
Weight (kg)^∗^	71.5 (±11.1)	53.3 (±7.1)	<0.001
Hand length (cm)^∗^	18.7 (±1.1)	17.5 (±0.7)	0.007
Thumb length (cm)^∗^	5.9 (±0.6)	5.4 (±0.5)	0.040
Smartphone experience (months)	36.8 (±13.6)	37.8 (±12.4)	1.000

*Values are mean (±SD). ^∗^Significant difference (p < 0.05) between male and female participants according to the results of Wilcoxon–Mann–Whitney test.*

### Experimental Design

Participants were assessed in single-task and DT conditions in a quiet corridor outside the research laboratory. This study adopted a 3×3 factorial design for both the task complexity (effect of the concurrent WM task; NW, DT with 1-back task, DT with 3-back task) and the road condition (Wide, Narrow, Obstacle) as within-subject factors. All participants were required to walk under each of the aforementioned nine conditions twice, and the administration order of the DT conditions was counterbalanced. The order of the NW tasks was randomized across participants and performed before the DTs.

For familiarization with the experimental procedures, after thorough briefing, individual participants practiced each level of the cognitive WM task for 20 min in a seated posture and then donned all the apparatus to walk on each road condition for 10 min while dual-tasking. The formal experimental session was performed at least 48 h after the familiarization session but within the time window of 96 h. Participants were asked to avoid sleep deprivation, strenuous exercise, and taking any new medication before the test.

After reporting to the laboratory during the day of the formal session, the participant had an extra 3 min to practice the cognitive tasks to avoid getting rusty. He or she donned the three-dimensional (3D) motion analysis system and the fNIRS system (to be described later), followed by conducting 60-s 1-back and 3-back WM tasks (to be described later) twice separately while standing still. Based on the predetermined order, the participant was then instructed to perform a series of gait tasks on different road conditions with or without concurrent cognitive tasks. All test trails had the identical time arrangement and consisted of one 5-s countdown block, one 60-s test block, and two 20-s quiet standing blocks before and immediately after the test block. During the quiet standing block, the participant was required to continuously fixate on the centered white cross shown on the black screen of the smartphone held by the right hand in portrait and remain as still as possible. Prior to the test block, a countdown of Arabic numerals was displayed on the screen, along with a reminder for the coming test contents. Depending on the assigned condition, in the following 60-s test block, the participant was required either to walk with the smartphone stored in the pouch (NW condition) or to perform the *n*-back task on it while walking (DT conditions). As this study aims to evaluate the effects of dual-tasking under more naturalistic scenarios, in all DT conditions, participants were not given explicit instruction about which particular task should be prioritized. After every two trials, there was a 2-min resting period during which the standard NASA-TLX questionnaire (Hart and Staveland, 1988) was administered to evaluate the perceived workload from the previous trial.

The gait tasks required participants to walk back and forth straight along a well-lit, stimulus-free 20-m walkway at a comfortable pace during the period of the test block. Three road conditions that represent common walking situations encountered in everyday life were examined.

• Wide: participants walked along a straight walkway 2 m in width.
• Narrow: a 0.3 m width straight walking area was outlined by red tape along the center of the same walkway. Participants were instructed to walk within the marked area at their preferred paces.
• Obstacle: five sets of obstacles, consisting of traffic cones 15 cm in height and 13 cm in depth connected side by side, were placed irregularly along the middle 18 m of the straight walkway. Participants were instructed to step over the obstacles without touching them. The locations of obstacles were pseudo-randomized, with the constraints that no two sets of obstacles were situated within 1 m. Moreover, one of the distances between two adjoined sets of obstacles was placed at least 6 m apart to make sure three continuous full gait cycles could be accommodated before obstacle negotiation occurred.

### Cognitive Working Memory Task

Variants of *n*-back paradigms have been employed by researchers to investigate neural activities related to WM in many neuroelectric and neuroimaging studies (Hoshi et al., 2003; Owen et al., 2005; Brouwer et al., 2012; Herff et al., 2013). In this study, computerized dual *n*-back WM tasks were programmed as an independent APP executed on the iPhone 4s (Apple, Cupertino, CA, USA). Participants had to monitor one verbal stimulus and one non-verbal stimulus presented simultaneously for every fixed period and react accordingly whenever the currently presented stimulus was the same as the one presented in the *n*-th period ago. The verbal stimulus (one of six different English letters) was considered as the target based on its auditory identity. The non-verbal stimulus (position) was considered as the target based on the location of an amber filled circle appearing in a three-by-three grid. As *n* increased, the complexity of the cognitive task also increased. The APP concurrently presented new stimuli for both types every 2500 ms. The stimuli appeared for the first 500 ms, and the screen showed the gird only for the remaining 2000 ms. Therefore, during the period of the 60-s test block involved in the cognitive task, a total of 24 verbal and 24 non-verbal stimuli were presented. Participants had to press either or both of the “sound” and “position” button at the bottom of the APP screen when they encountered the target(s). The participants were instructed to perform the *n*-back WM tasks as accurately as possible during the trials. The ratio of the number of correct responses to the number of all responses was employed as the accuracy indicator for performance measures, along with the average RT of the correct responses.

### Assessments of Gait Kinematics

**FIGURE 1:**
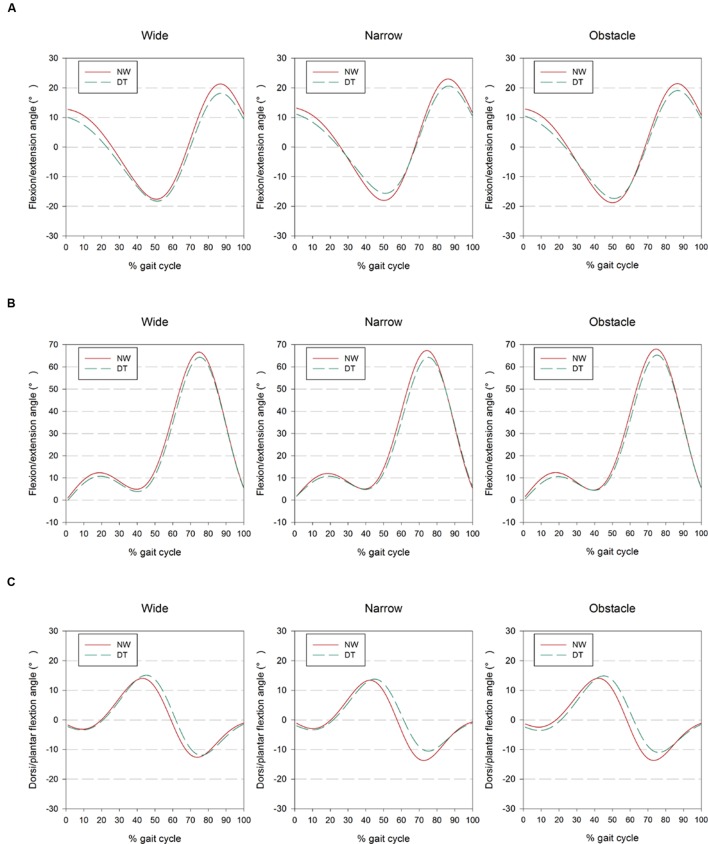
**Presentation of (A) hip, (B) knee, and (C) ankle joint kinematics from the right side of 24 participants as ensemble-averaged waveforms for different combinations of cognitive task complexity and walking road condition**. The results obtained from 1-back and 3-back task are combined and showed as DT for clarity.

For locomotion registration, a commercially available 3D motion analysis system, Xsens MVN BIOMECH (Xsens Technologies BV, Enschede, Netherlands), with a sampling rate of 120 Hz, was used to measure lower body kinematics while the participants performed over-ground walking in different road conditions. Seventeen magnetic and inertial measurement units were attached to associated body segments using Velcro straps in accordance with the Xsens user manual. Functional calibrations were employed to establish the transformation matrix between the local sensor frame and the anatomical frame of the associated body segment to which the sensor was attached (Favre et al., 2009). Subsequently, 3D joint waveforms following the coordination system defined by the International Society of Biomechanics were calculated using proprietary algorithms and individual anatomical measurements (Roetenberg et al., 2009). The system has demonstrated a high within-day and between-day repeatability, as well as good agreement with other camera-based motion capture systems, in 3D joint angle kinematic measurements (Favre et al., 2009; Cloete and Scheffer, 2010; Zhang et al., 2013). Data acquisition and synchronization with the fNIRS system were performed via the Awinda/Sync station and the software interface of MVN Studio Pro (v.3.5.1, Xsens Technologies BV, Enschede, Netherlands). Kinematic data were low-pass filtered at 10 Hz using a zero-lag fourth order Butterworth filter. The timings of initial contact and toe-off were determined using the algorithms developed for gait event detection (Zijlstra and Hof, 2003; Jasiewicz et al., 2006; Gonzalez et al., 2010) and visually inspected using the video recordings from the synchronized MVN Ethernet camera. Kinematic data from three consecutive full gait cycles (determined from initial contacts of the right foot), occurring either in the middle of the 20-m straight corridor (for the Wide and Narrow road conditions) or immediately before the obstacle negotiation (for the Obstacle road condition), were time-normalized to the percent gait cycle. Based on these gait events, spatiotemporal gait parameters (e.g., gait speed, stride time, and step length) were calculated using a custom MATLAB script (v 2014b, The MathWorks, Inc., Natick, MA, USA) and normalized with respect to individual stature (% SL) when adequate. The stride-by-stride variability levels of these gait parameters were also quantified using the CV as measures of gait inconsistency associated with executive function. To estimate the extent of kinematic adaptation for postural control, average and peak values of hip, knee, and ankle joint movement in the sagittal plane were calculated from the ensemble-averaged curves of the consecutive gait cycles (as shown in **Figure 1**), along with the corresponding standard deviation (SD), to quantify joint variability.

### Assessment of Prefrontal Lobe Neural Activation

Cerebral neural activation was assessed by a portable fNIRS system (PortaLite, Artinis, Netherlands) through neurovascular coupling mechanisms. Because HbO and HbR have distinct spectral absorption properties in the near-infrared region, relative changes in the concentration of each hemoglobin component can be quantitatively estimated by the modified Beer-Lambert law (Miyazawa et al., 2013). The fNIRS head probe with three emitters (with light wavelengths of 760 nm and 850 nm) and one detector was tightly secured over the right PFC region (Fp2) of the participant’s forehead, according to the international EEG 10–20 system (Klem et al., 1999). The raw NIRS intensity data were continuously recorded at 50 Hz in all three channels during the test trials. Subsequently, the estimated HbO and HbR waveforms from the channel with a 40 mm emitter–detector separation were chosen (Miyazawa et al., 2013) and then further low-pass filtered with a finite impulse response filter with a cut-off frequency of 0.2 Hz to attenuate the noises from non-evoked (spontaneous) neurovascular coupling (Scholkmann et al., 2014). Because the fNIRS measurement depth is roughly half the distance between the emitter and detector optode, the measured hemodynamic activities were mainly from the FPC, in accordance with the superficial BA 10. In this study, we focused on the measured change in HbO concentration (ΔHbO), as it has shown a slightly better correlation with fMRI BOLD signals (Cui et al., 2011) and higher sensitivity to locomotion-dependent changes in regional cerebral blood flow (Miyai et al., 2001; Harada et al., 2009; Holtzer et al., 2011; Scholkmann et al., 2014). The ΔHbO waveform recorded during each test trial was offset to the baseline value obtained at the beginning of the quiet standing block. To compare cerebral activation across distinct levels of cognitive task complexity and road condition, the mean, 10th percentile, 90th percentile, and range of variation (the difference between the 10th and 90th percentile values) of ΔHbO waveforms during the period of the 60-s test block were examined separately in the associated statistical analyses.

### Statistical Analyses

All the following statistical analyses were conducted in SAS (v9.4, SAS Institute, Cary, NC, USA). Normal probability plots for the examined outcome measures were first constructed individually to identify the data distributions. Box–cox power transformations were then performed for the measures whose data deviated substantially from normality. To verify whether the classification of cognitive task complexity and road condition induced different perceived workload during test trails, the NASA-TLX score was analyzed with rANOVA using linear mixed modeling approaches. The within-subject factors were the complexity of cognitive task (Task), with three levels NW, 1-back, and 3-back), and the road condition (Road) with three levels (Wide, Narrow, Obstacle). The administration order of task conditions and the interaction terms with Task and Road factors were treated as covariates to control its influence by reducing error variance. To assess the potential gender effect, we also had Gender as a between-subject factor. Consequently, in the rANOVA, the aforementioned factors were considered as fixed effects, with Participant modeled as a random effect. Both the unrestricted covariance structure and component variance structure for repeated measures were fitted separately for the same dependent variable. The likelihood ratio test statistics and corresponding *p*-value were then calculated using the –2REML log-likelihood values from the rANOVA results of two different fitted covariance structures. The rANOVA model with a more parsimonious covariance structure (i.e., component variance one) was chosen for further inference if the *p*-value of the likelihood ratio test was higher than 0.05 (West et al., 2014). For the omnibus *F* tests, any result with *p*-value < 0.05 was considered statistically significant. *Post hoc* multiple comparison tests using the Tukey–Kramer adjustment were conducted between levels of the factors if necessary.

Subsequently, the associated outcomes of *n*-back task performances, low-extremity joint kinematic data, and spatiotemporal gait parameters were analyzed separately using the same rANOVA modeling procedures mentioned above. For fNIRS data, to examine the influence of time, the waveforms of changes in HbO concentration during the 60-s test blocks were further subdivided into three 20-s time periods. Therefore, 3 (complexity of cognitive Task) × 3 (Road condition) × 2 (Gender) × 3 (Time: 25–45 s, 45–65 s, and 65–85 s of the whole test trial) rANOVA was used to analyzed the levels and variability of ΔHbO. We also calculated Pearson’s bivariate correlation to examine the perceived task workload, the performance outcomes of the *n*-back task, and their associations with locomotion (gait speed, step length, and stride time) and neural activation (HbO) of the PFC.

## Results

As expected, the NASA-TLX score differed significantly across various levels of cognitive task complexity (*F*_2,288_= 131.3, *p* < 0.001) and road condition (*F*_2,288_= 30.8, *p* < 0.001). The NW condition had the lowest TLX score (24.4 ± 2.1), followed by DT of the 1-back task (27.9 ± 1.5) and DT of the 3-back task (41.6 ± 1.7), with all pair comparisons reaching the significant level (*p* < 0.001 to *p* = 0.015). Moreover, walking on the wide road also resulted in a substantially lower NASA-TLX score (27.9 ± 1.8) than either walking on the narrow (35.3 ± 1.9, *p* < 0.001) or obstacle road (34.8 ± 1.8, *p* < 0.001). The results confirmed that the experimental conditions examined in this study in terms of cognitive task complexity and road condition were sufficient to elicit distinct perceived task workloads for the participants.

### Performance Measures of the Working Memory Task

**FIGURE 2:**
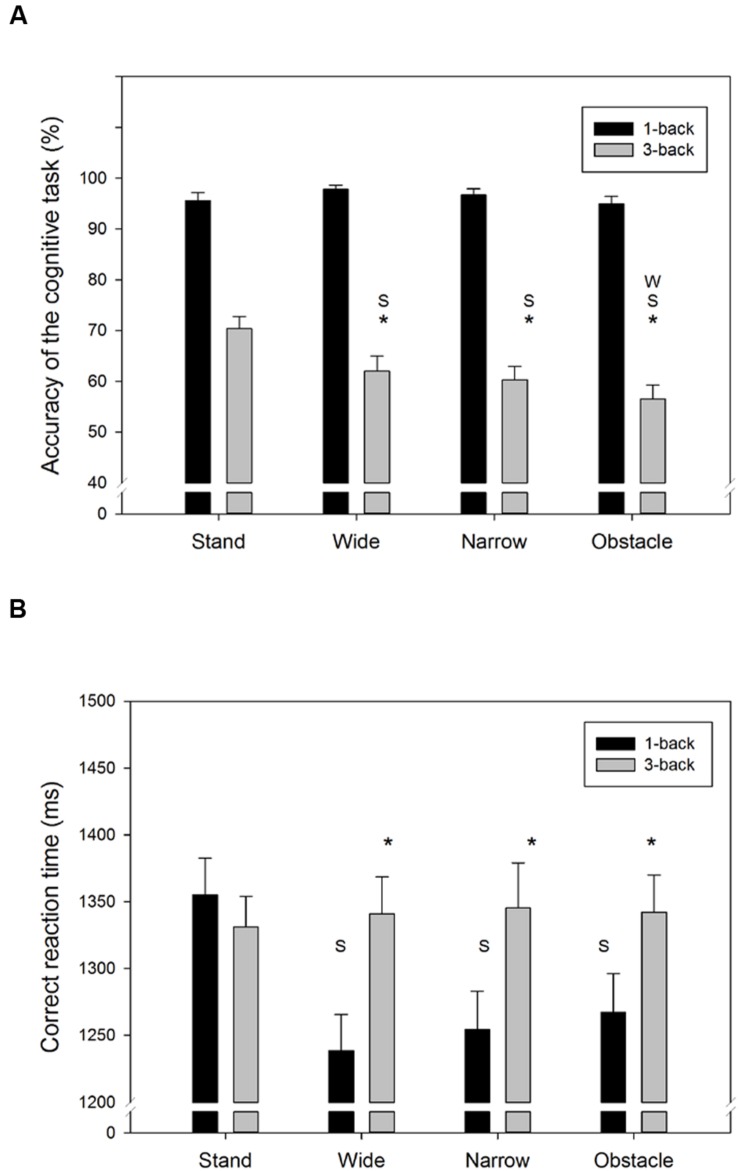
**Summary behavior outcomes of the ***n***-back WM tasks for (A) accuracy, and (B) correct RT in all combinations of cognitive task complexity and walking road condition**. Error bars represent ±1 SE. An asterisk (^∗^) denotes a significant difference (*p* < 0.05) between the 1-back and 3-back task under the same walking road condition. Under the same cognitive task complexity, a significant difference (*p* < 0.05) between the target road condition and the stand/wide/narrow road condition is denoted by the symbol S/W/N, respectively.

**Figure 2** showed the accuracy and RT of the *n*-back WM tasks from the single- and DT walking conditions. The average response of the 3-back task was considerably less accurate (*F*_1,318_= 708.0, *p* < 0.001) and slower (*F*_1,318_= 21.1, *p* < 0.001) than for the 1-back task. Moreover, compared to the standing still condition, walking while concurrently conducting the *n*-back tasks also tended to decrease the task accuracy (*F*_3,318_= 6.6, *p* < 0.001) but, on average, resulted in shorter response times (*F*_3,318_= 3.2, *p* = 0.025). However, as seen in **Figures 2A,B**, there were significant Task by Road interactions for both the accuracy (*F*_3,318_= 6.7, *p* < 0.001) and RT (*F*_3,318_= 4.7, *p* = 0.003) measures. For instance, in the DT 1-back conditions, a trend of increase in RT corresponding to the difficulty level of the road conditions (Wide < Narrow/Obstacle, as indicated by the NASA-TLX score) was observed, but was absent in the DT 3-back conditions (**Figure 2B**). The aforementioned differences across road conditions, however, did not reach the level of significance in the *post hoc* analyses.

### Spatio-Temporal Gait Characteristics

**FIGURE 3:**
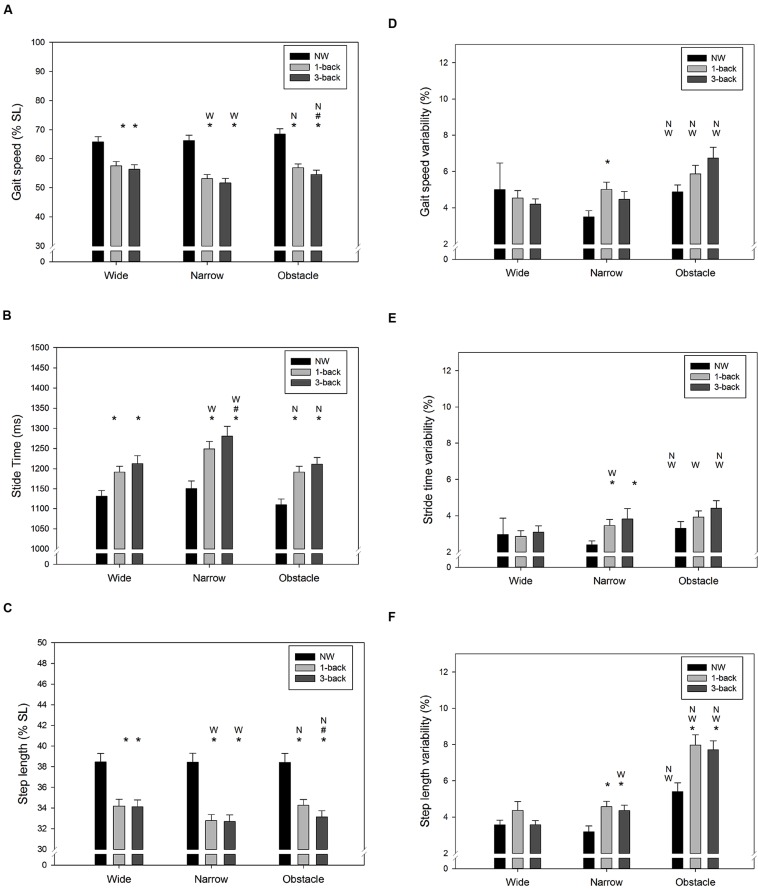
**Summary results of spatiotemporal gait parameters for (A) average gait speed, (B) average stride time, (C) average step length, (D) variability of gait speed, (E) variability of stride time, and (F) variability of step length in all combinations of cognitive task complexity and walking road condition**. Error bars represent ±1 SE. Under the same walking road condition, a significant difference (*p* < 0.05) between the target cognitive task complexity and l-back/3-back task is denoted by the symbol asterisk (^∗^)/pound (#), respectively. Under the same cognitive task complexity, a significant difference (*p* < 0.05) between the target road condition and the wide/narrow road condition is denoted by the symbol W/N, respectively.

As expected, there were significant Task and Road effects on gait speed (Task: *F*_2,288_= 140.4, *p* < 0.001; Road: *F*_2,288_= 10.0, *p* < 0.001), stride time (Task: *F*_2,288_= 60.4, *p* < 0.001; Road: *F*_2,288_= 25.4, *p* < 0.001), and step length (Task: *F*_2,288_= 158.1, *p* < 0.001; Road: *F*_2,288_= 5.7, *p* = 0.004). The NW condition resulted in the fastest gait speed and the shortest stride time, followed by the 1-back DT condition and 3-back DT condition, respectively (**Figures 3A,B**). The average step length of the walk-only condition was also significantly longer than when *n*-back tasks were performed concurrently (both *p* < 0.001, **Figure 3C**). Compared to the wide road and the obstacle road, walking on the narrow road generally lead to a slower gait speed, a longer stride time, and a shorter step length, as shown in **Figures 3A–C**. Similarly, the CV of gait speed (Task: *F*_2,288_= 4.5, *p* = 0.012; Road: *F*_2,288_= 20.1, *p* < 0.001), stride time (Task: *F*_2,288_= 8.0, *p* < 0.001; Road: *F*_2,288_= 16.7, *p* < 0.001), and step length (Task: *F*_2,288_= 13.5, *p* < 0.001; Road: *F*_2,288_= 77.0, *p* < 0.001) varied across the different Task and Road conditions examined. Either dual-tasking or walking on the obstacle road resulted in higher variability (less consistency) in gait speed (**Figure 3D**), stride time (**Figure 3E**), and in step length (**Figure 3F**) than their counterparts. It was noteworthy that the average stride time was found to be moderately correlated with the NASA-TLX score (*r* = 0.31, *p* < 0.001), whereas the gait speed was inversely correlated with the perceived workload level (*r* = –0.22, *p* < 0.001). Furthermore, a slight positive correlation between the CV of stride time and the NASA-TLX score (*r* = 0.15, *p* = 0.012) was observed in our data.

### Coordination of the Lower-Extremity Joint Motion

**Table 2** summarizes how the participants coordinated their lower body joint angles in the sagittal plane to accommodate the concurrent performance of the WM tasks while walking on different road conditions. It can be seen that compared with the kinematic data measured from the consecutive gait cycles, the hip, knee, and ankle joints all demonstrated significantly different peak angles (Max/Min value) in either flexion or extension direction, as well as angle variability (SD) and mean values over the gait cycles, among the various Task and Road conditions (*p* < 0.001 to *p* = 0.01). Moreover, significant Task by Road interactions were also found in the Min and SD measures of the hip and ankle joints.

**Table 2:** Summary of lower-extremity joint kinematics in the sagittal plane for rANOVA main effects and *p*-values for the interactions^1,2^.

		Hip flexion				Knee flexion				Ankle dorsiflexion			
		Mean	Max	Min^3^	*SD*	Mean	Max	Min^3^	*SD*	Mean	Max	Min^3^	*SD*
Cognitive task	Dof	2,288	2,288	2,288	2,288	2,288	2,288	2,288	2,288	2,288	2,288	2,288	2,288
	*F* ratio	12.07	36.29	8.16	79.17	28.17	29.44	4.69	20.42	13.85	6.52	36.36	13.24
	*p*-value	**<0.001**	**<0.001**	**<0.001**	**<0.001**	**<0.001**	**<0.001**	**0.01**	**<0.001**	**<0.001**	**0.002**	**<0.001**	**<0.001**
	NW	2.3 (0.5)^A^	22.0 (0.5)^A^	–18.3 (0.5)^B^	13.3 (0.2)^A^	25.2 (0.5)^A^	67.6 (0.7)^A^	0.7 (0.4)^A^	22.1 (0.2)^A^	−0.3 (0.4)^B^	14.4 (0.5)^B^	−13.9 (0.6)^B^	8.6 (0.1)^A^
	1-back	1.1 (0.3)^B^	19.5 (0.3)^B^	–17.3 (0.3)^A^	12.2 (0.1)^B^	23.8 (0.4)^B^	65.4 (0.5)^B^	0.2 (0.3)^B^	21.6 (0.1)^B^	0.4 (0.3)^A^	15.1 (0.3)^A^	−11.99 (0.5)^A^	8.4 (0.1)^B^
	3-back	1.2 (0.3)^B^	19.3 (0.4)^B^	–17.0 (0.3)^A^	11.9 (0.1)^B^	23.4 (0.4)^B^	64.4 (0.5)^C^	0.3 (0.3)^B^	21.3 (0.2)^C^	0.5 (0.3)^A^	14.9 (0.3)^A^	−11.5 (0.4)^A^	8.2 (0.1)^B^

Road condition	Dof	2,288	2,288	2,288	2,288	2,288	2,288	2,288	2,288	2,288	2,288	2,288	2,288
	*F* ratio	22.2	25.59	16.2	0.56	3.58	4.42	12.21	6.40	2.65	19.97	1.67	31.01
	*p*-value	**<0.001**	**<0.001**	<0.001	0.57	**0.029**	**0.013**	**<0.001**	**0.002**	0.073	**<0.001**	0.189	**<0.001**
	Wide	0.6 (0.3)^B^	18.9 (0.4)^B^	–18.2 (0.3)^B^	12.2 (0.1)^A^	23.6 (0.4)^B^	65.0 (0.5)^B^	−0.3 (0.3)^B^	21.5 (0.2)^B^	0.3 (0.2)^A^	15.3 (0.4)^A^	−12.6 (0.5)^A^	8.7 (0.1)^A^
	Narrow	2.4 (0.3)^A^	21.2 (0.4)^A^	–16.2 (0.4)^A^	12.2 (0.2)^A^	24.1 (0.4)^AB^	65.3 (0.6)^AB^	1.0 (0.3)^A^	21.4 (0.2)^B^	0.2 (0.3)^A^	14.2 (0.4)^B^	−11.8 (0.5)^A^	8.0 (0.1)^B^
	Obstacle	1.1 (0.3)^B^	19.7 (0.4)^B^	–17.7 (0.4)^B^	12.3 (0.1)^A^	24.1 (0.4)^A^	66.1 (0.6)^A^	0.2 (0.3)^B^	21.8 (0.2)^A^	0.4 (0.3)^A^	15.1 (0.4)^A^	−12.0 (0.5)^A^	8.5 (0.1)^A^

Interactions	Dof	4,288	4,288	4,288	4,288	4,288	4,288	4,288	4,288	4,288	4,288	2,288	4,288
	*F* ratio	2.2	0.44	4.21	3.58	0.72	0.87	0.87	1.82	1.36	1.49	4.02	5.70
	*p*-value	0.0693	0.777	**0.003**	**0.007**	0.577	0.6	0.4845	0.125	0.2491	15.1	**0.003**	**<0.001**

^1^*Values are presented as mean (SE) in degree unit. Repeated measures ANOVA with participants as a random effect and cognitive task, road condition as fixed effects. ^2^The groupings based on the post hoc analyses are indicated by the superscript and ranked in a descending order such as A > B > C. The same superscript means no significant difference. ^3^Negative values of the minimum gait parameter represent hip extension, knee extension, and ankle plantar-flexion, respectively. All p-value numbers smaller than 0.05 are bold.*

Generally, the data suggested that participants tended to both flex and extend their hips less during dual-tasking than when walking only (*p* < 0.001 to *p* = 0.011). We observed the smallest peak values of knee flexion in the DT 3-back condition, followed by the values from the DT 1-back condition and from the NW condition, respectively (**Table 2**). Meanwhile, walking and concurrently conducting the *n*-back tasks caused the participants to flex their ankles significantly further toward the dorsal side of the foot but less toward the plantar side (*p* < 0.001 to *p* = 0.014). In comparison with NW, the participants varied all their joint angles less frequently over the time course of the gait cycle when dual-tasking with the *n*-back tasks (*p* < 0.001 to *p* = 0.002), as indicated by the SD measure in **Table 2**. Moreover, in the knee joint, we further observed a smaller peak flexion (*p* = 0.009) and a decreased SD (*p* = 0.008) when dual-tasking with the 3-back task than with the 1-back task.

Regarding the comparisons among road conditions, participants were found to flex their hips significantly further but extend them less while walking on the narrow road than on the wide or obstacle roads (all *p* < 0.001; **Table 2**, also shown in **Figure 1**). Among the three road conditions, the narrow road condition, on average, also had the smallest peak value of ankle dorsiflexion, whereas no difference was found in the plantar-flexion direction. We further observed the tendency of participants to flex their knees to a greater extent before negotiating the obstacle than when walking freely on the wide road (*p* = 0.012). Moreover, the statistical results in **Table 2** also suggested that walking on the narrow road led to a slightly smaller SD in the knee and ankle joints than walking on either the wide or obstacle road.

### Hemodynamic Activities in the Frontopolar Cortex

**FIGURE 4:**
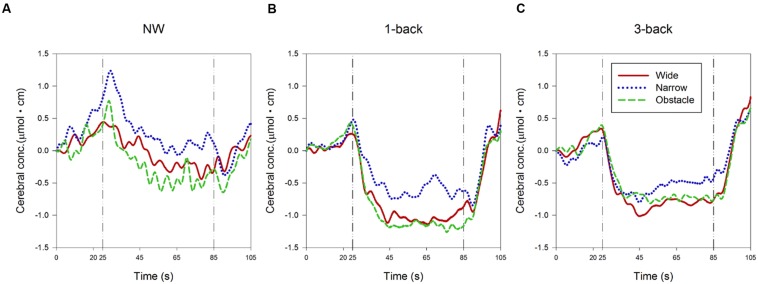
**Presentation of changes in the relative concentration level of HbO during (A) normal walking, (B) dual-tasking with the 1-back task, and (C) dual-tasking with the 3-back task from pre-test quiet standing (0–20 s), countdown block (20–25 s), test block (25–85 s), and post-test quiet standing (85–105 s)**. Graphs are showed as ensemble-averaged waveforms from 24 participants for different combinations of cognitive task complexity and walking road condition.

The ensemble-averaged waveforms of the relative concentration change of HbO (ΔHbO) over the time course of the 60-s experiment period for each condition are depicted in **Figure 4**. In the NW condition (**Figure 4A**), a substantial increase in ΔHbO was observed from the beginning of the quiet-standing preparation period to the onset of walking, followed by a gradual decrease in ΔHbO throughout the first two-thirds of the test block period. On the other hand, when the participants were required to concurrently perform the 1-back WM task while walking, the ΔHbO showed a trend of increasing only after the 5-s countdown started. However, a much larger and steeper decrease in ΔHbO occurred immediately while dual-tasking began. Consequently, the ΔHbO waveforms descended to their plateau approximately 20 s earlier than in the NW condition (**Figure 4B**). In dual-tasking with the 3-back task, the general patterns of ΔHbO waveforms in **Figure 4C** were similar to the ones shown in the DT 1-back condition, albeit with relatively higher levels of waveform plateau, especially in the waveforms measured from participants walking on the wide or obstacle road.

As expected, the statistical results of rANOVA in **Table 3** indicated that ΔHbO changed significantly over time in all waveform outcomes (all *p* < 0.001). During the period of 25–45 s (the first one-third of the test block), the participants’ FPC, on average, exhibited a higher mean level and wider range of variation in the measured ΔHbO compared to the later two-thirds of the test block. Nonetheless, no difference in any HbO outcomes between the second and the third 20-s walking periods was considered significant. Both the main effects of “complexity of cognitive Task” and “Road condition” were significant in the associated rANOVA for the examined measures of ΔHbO waveforms (with *p* < 0.001 to *p* = 0.004 in **Table 3**). Subsequent *post hoc* analyses further revealed that the mean, 10th, and 90th percentile levels of ΔHbO in the DT conditions were all lower than in the NW condition, with the largest reduction in hemodynamic responses found in the DT 1-back condition (**Figure 4**). However, a considerably wider range of ΔHbO fluctuation was also observed when the participants walked without performing any *n*-back tasks (NW vs. 1-back, *p* < 0.001; NW vs. 3-back, *p* < 0.001).

**Table 3:** Summary of changes in oxygenated hemoglobin concentration in the prefrontal pole area for rANOVA main effects and *p*-values for the interactions^1,2^.

		Δ HbO concentration			
		Mean	P10	P90	Range
Cognitive task	Dof	2,998	2,998	2,998	2,998
	*F* ratio	56.18	22.41	47.99	33.50
	*p*-value	**<0.001**	**<0.001**	**<0.001**	**<0.001**
	NW	−0.16 (0.07)^A^	−0.81 (0.08)^A^	0.53 (0.08)^A^	1.34 (0.06)^A^
	1-back	−0.90 (0.06)^C^	−1.40 (0.06)^C^	−0.31 (0.06)^C^	1.10 (0.05)^B^
	3-back	−0.65 (0.06)^B^	−1.12 (0.06)^B^	−0.10 (0.06)^B^	1.02 (0.03)^B^

Road condition	Dof	2,998	2,998	2,998	2,998
	*F* ratio	17.99	23.38	12.61	5.45
	*p*-value	**<0.001**	**<0.001**	**<0.001**	**0.004**
	Wide	−0.72 (0.05)^B^	−1.21 (0.06)^B^	−0.15 (0.07)^B^	1.06 (0.04)^B^
	Narrow	−0.43 (0.06)^A^	−0.95 (0.07)^A^	0.17 (0.07)^A^	1.11 (0.05)^B^
	Obstacle	−0.81 (0.07)^B^	−1.35 (0.07)^C^	−0.18 (0.07)^B^	1.17 (0.04)^A^

Time	Dof	2,998	2,998	2,998	2,998
	*F* ratio	24.96	11.08	77.50	106.03
	*p*-value	**<0.001**	**<0.001**	**<0.001**	**<0.001**
	25-45 s	−0.41 (0.05)^A^	−1.06 (0.06)^A^	0.44 (0.05)^A^	1.50 (0.05)^A^
	45-65 s	−0.79(0.07)^B^	−1.24 (0.07)^B^	−0.32 (0.07)^B^	0.92 (0.03)^B^
	65-85 s	−0.76 (0.07)^B^	−1.21 (0.07)^B^	−0.28 (0.08)^B^	0.93 (0.05)^B^

Interactions				
Task × Road	Dof	4,998	4,998	4,998	4,998
	*F* ratio	2.25	2.76	1.31	1.17
	*p*-value	0.062	**0.027**	0.264	0.322
Task × Time	Dof	4,998	4,998	4,998	4,998
	*F* ratio	1.88	2.92	0.83	4.31
	*p*-value	0.112	**0.021**	0.504	**0.002**
Task × Time	Dof	4,998	4,998	4,998	4,998
	*F* ratio	0.42	0.43	0.76	0.29
	*p*-value	0.794	0.784	0.550	0.886

^1^*Values are presented as mean (SE) in μmol.cm unit. Repeated measures ANOVA with participants as a random effect and cognitive task, road condition, and time as fixed effects. ^2^For each dependent variable, the groupings based on the post hoc analyses are indicated by the superscript and ranked in a descending order such as A > B > C. The same superscript means no significant difference. All p-value numbers smaller than 0.05 are bold.*

Regarding the influences of road condition on the neural processes, we found that the ΔHbO waveforms from the wide and obstacle road condition generally increased less, decreased more, and had a significantly lower mean level of ΔHbO compared to the narrow road condition (**Table 3** and **Figure 4**). Moreover, the obstacle road condition also showed a wider range of ΔHbO during the walking test block than either the wide (*p* = 0.005) or the narrow road condition (*p* = 0.038). It should be noted that the “Task” by “Road” interaction in the 10th percentile level measure did reach the significant level (*p* = 0.027). Further examination implied that in both the NW (**Figure 4A**) and the DT 1-back condition (**Figure 4B**), walking on the obstacle road led to even lower ΔHbO waveforms than walking on the wide road (with *p* = 0.002 and 0.015, respectively). The aforementioned observation, however, was not obvious in the DT 3-back condition (*p* = 0.654; **Figure 4C**). A highly significant “Task” by “Time” interaction (*p* = 0.002) in the range measure, in contrast, reflected greater fluctuation in the ΔHbO waveform of the NW condition during the 45th to 85th seconds of the trail time, compared to the DT conditions, as shown in **Figure 4**.

### Correlations between Behavior Outcomes and Concentration Changes in Oxygenated Hemoglobin

No significant correlation between the RT and the accuracy of the *b*-back tasks and fNIRS data were found in our data. Changes in the subjectively measured task workload did significantly change with the fNIRS data, although the magnitude of the correlations varied across the road conditions examined. In the obstacle and narrow road conditions, a higher NASA-TLX score was generally linked to a higher level of ΔHbO in terms of mean (obstacle: *r* = 0.33, *p* < 0.001; narrow: *r* = 0.24, *p* = 0.007), 10th percentile (obstacle: *r* = 0.33, *p* < 0.001; narrow: *r* = 0.25, *p* = 0.006), and 90th percentile (obstacle: *r* = 0.26, *p* = 0.004; narrow: no significant, *p* = 0.087). A higher value of the NASA-TLX score was also associated with a reduced range of ΔHbO variation in the obstacle road (*r* = –0.33, *p* < 0.001) and the narrow road condition (*r* = –0.23, *p* = 0.011). Nevertheless, the aforementioned association between the perceived workload and ΔHbO outcomes was not observed in the wide road condition.

## Discussion

This study used fNIRS and a 3-D movement analysis system to investigate the neutral correlates of DT walking on the postural control and executive function in healthy young individuals. Specifically, the influences of cognitive task complexity and walking road condition were quantified comprehensively using behavioral outcomes, gait kinematics, and hemodynamic activation in the FPC and their associations during cognitive–motor interaction. Our results suggested that compared to the single-task condition, the task workload perceived by healthy younger adults was not only higher under the DT conditions but was further increased as the cognitive task and road conditions became more challenging. Moreover, the additional demands on executive functions and postural control were found to significantly affect neural activation in the participants’ PFC and lower-extremity joint kinematics during ambulation. Collectively, walking while performing a concurrent WM task led to decreased cognitive task performance, modified gait characteristics, and more importantly, noticeably increased gait variability. Moreover, some of these DTC became more profound when walking in challenging road conditions.

### Behavioral Findings

The current results showed that no difference in task accuracy was found between DT walking and standing still when the easier dual 1-back task was conducted. Mirelman et al. (2014) also found similar rates of task completion and mistakes in the examined serial seven subtraction task, regardless of whether their healthy young participants were walking or not. On the other hand, we observed a significantly lower accuracy of the concurrent dual 3-back task in the DT walking condition than in a standing condition, whereas the resulting RT obtained from the two conditions were similar (**Figure 2**). To some extent, our results confirmed the findings of past studies in which the performance decrement of the concurrent non-gait task tends to become more profound when the secondary task requires ongoing visual observation (Bock, 2008; Beurskens and Bock, 2013; Beurskens et al., 2014) or likely causes more motor-cognitive interference, such as texting (Plummer et al., 2015).

### Spatiotemporal Gait Parameters

Under DT walking, our young participants exhibited a lower gait speed, accompanied by a lengthened stride time and a reduced step length compared to NW. Moreover, the increased WM load of the concurrent cognitive task further exacerbated the changes in the aforementioned spatiotemporal gait characteristics (**Figures 3A–C**). Similar findings have been reported in the literature (Yogev-Seligmann et al., 2010; Al-Yahya et al., 2011; Beurskens and Bock, 2013; Beurskens et al., 2014; Schabrun et al., 2014; Agostini et al., 2015; Plummer et al., 2015), although the magnitude of DTC in particular gait parameters varied among studies due to differences in the non-gait tasks utilized and in the experimental setups. For instance, reductions of gait speed by 19–24% have been reported by studies that required participants to either type a pre-assigned sentence (Schabrun et al., 2014) or phrases appearing on the smartphone screen (Plummer et al., 2015) while walking. In contrast, a much smaller decrease in velocity (12%) was observed when the concurrent task used (e.g., VFT) did not demand additional regulation of postural control (Yogev-Seligmann et al., 2010). Therefore, the average 18% DT decrement in gait speed demonstrated in this study was not unexpected given that the participants had to hold a smartphone to conduct the *n*-back WM task while walking.

Gait variability has been considered as a promising measure for characterizing balance control and may be more sensitive to age-related impairments in DT walking than mean-based spatiotemporal gait measures (Springer et al., 2006; Priest et al., 2008; Schrager et al., 2008; Yogev-Seligmann et al., 2010; Al-Yahya et al., 2011; Beurskens and Bock, 2013). Our results showed that compared to walking normally, the variability of gait speed, stride time, and step length all increased significantly when young individuals had to simultaneously perform attention-demanding *n*-back tasks while walking. These results are consistent with the findings from previous research that used smartphone texting (Agostini et al., 2015) or VFT (Yogev-Seligmann et al., 2010) as the concurrent non-gait task. In contrast, walking while performing mental arithmetic (Springer et al., 2006; Mirelman et al., 2014; Francis et al., 2015) or a simple sensorimotor task (Beurskens and Bock, 2013) seems have little influence on gait variability in healthy young adults. Therefore, it could be argued that some non-gait tasks may not be challenging enough to disrupt the central regulation of periodic locomotion patterns.

In addition to evaluating the effect of cognitive task demand on DT walking, the current study further examined the impact of walking condition on gait control. Consistently with previous studies (Schrager et al., 2008; Francis et al., 2015), we did not observe significant difference in gait variability between wide and narrow road conditions when no secondary task was involved. However, while dual-tasking in the narrow road condition our participants were found to reduce their step length and increase stride time, thus resulting in a slower gait speed than in the other two road conditions (**Figures 3A–C**). These findings further extend the knowledge on obstacle negotiation behavior in healthy young adults by showing that compared to unobstructed walking, the additional motor coordination required while approaching obstacles could significantly destabilize gait patterns, regardless of whether they were performing a concurrent WM task or not (**Figures 3D–F**). Recently, Beurskens and Bock (2013) also observed a significant increase in step duration variability while young adults walked on a narrow path or avoided obstacles during a visual checking task. Considering that the dual *n*-back task used in the current study also required participants to visually interact with the smartphone continuously, the observed DTC in gait variability may be partially explained by the disruption of on-line visual feedback, which is crucial for adaptive human locomotion (Patla and Greig, 2006).

### Lower-Extremity Joint Kinematics

The bilateral coordination of limb movements is known to be susceptible to the cognitive workload imposed during DT walking (Yogev-Seligmann et al., 2013). The impact of dual-tasking on locomotion has been primarily quantified in terms of changes in spatiotemporal gait parameters in previous works (Springer et al., 2006; Schrager et al., 2008; Yogev-Seligmann et al., 2010; Al-Yahya et al., 2011; Beurskens and Bock, 2013; Beurskens et al., 2014; Mirelman et al., 2014; Francis et al., 2015; Plummer et al., 2015). However, these measures do not directly reveal which specific kinematic adaptations individuals adopt to maintain dynamic balance when walking on different road conditions (Dingwell and Marin, 2006; Perry and Burnfield, 2010). The data presented in this study (**Figure 1** and **Table 2**) demonstrated that healthy young adults significantly altered their joint kinematics to accommodate the motor-cognitive interference that resulted from DT walking on different road conditions. The adjustments included reduced peak hip angle in both the flexion and extension directions, along with a decrease in knee flexion and ankle plantarflexion. Collectively, these subtle but significant changes within gait cycles led to a shorter step length and a longer stride time, which may reflect the participants’ inclination to spend more time with both feet supported by the ground. These particular adaptations exhibited by our participants could, in turn, curtail the additional demand for postural control (Kelly et al., 2008), and at the same time facilitate the performance of *n*-back tasks by maintaining the smartphone in a steady position in the visual field (Schabrun et al., 2014). It should be noted that the recent work of Agostini et al. (2015) did not reveal any significant kinematic changes in knee and angle joints during walking and texting compared to NW. These disparate results could be partially explained by the differences in experimental methodologies and instrumentation between the two studies. In addition to the influence of the concurrent cognitive task, the presented data also illustrated varied kinematic strategies in response to different road conditions. Young individuals were found to extend their hip joints further in the flexion direction but reduced their peak hip extension when walking in the narrow condition. These particular alterations were not surprising, as the individuals exhibited a tendency to place their feet in tandem to avoid stepping over the demarcated boundary, resulting in reductions in step length and gait speed, an observation consistent with the findings in prior research (Kelly et al., 2008; Schrager et al., 2008).

### Neural Activation

Our hypothesis that dual-tasking would increase the demands of central executive function and affect the patterns of neural activation in the PFC was partially supported by the experimental results. Consistently with recent studies (Holtzer et al., 2011; Doi et al., 2013; Beurskens et al., 2014; Mirelman et al., 2014), we observed significant differences in the relative changes in HbO concentration levels between NW and walking while performing a concurrent secondary task (**Table 3**). Moreover, the observed ΔHbO waveforms registered during DT walking demonstrated distinguishable patterns of prefrontal engagement in response to the varied difficulty levels of the *n*-back task (**Figure 4**). These conclusions were consistent with the findings obtained from neuroimaging studies of WM, in which activation in the lateral/medial premotor cortex, dorsolateral/ventrolateral PFC, FPC, and lateral/medial posterior parietal cortex have been consistently reported with variants of *n*-back paradigms (Hoshi et al., 2003; Owen et al., 2005; Herff et al., 2013). Having healthy young adults perform walking while counting forward and walking while reciting serial subtractions by seven, Mirelman et al. (2014) also found that the two conditions induced distinct HbO levels in BA10. Moreover, the present data suggest that young individuals walking on different road conditions would exhibit dissimilar ΔHbO levels throughout the testing period, especially in dual-tasking (**Figure 4** and **Table 3**). Although it is known that locomotion can require inputs from higher-level cognitive function and sometimes demand considerable attention resources for the coordination of bilateral limb movements (Mihara et al., 2007; Harada et al., 2009; Yogev-Seligmann et al., 2013), to the best of our knowledge, this work was the first fNIRS study that quantified the influence of road conditions on the central executive function during DT overground walking. Our findings, therefore, further support the evidence obtained from past fMRI studies (Wang et al., 2009; Wai et al., 2012; van der Meulen et al., 2014) that have found the complexity of road condition (e.g., irregular path, obstacle negotiation) to lead to differential neural responses in the PFC, SMA, visual regions, parietal lobule, primary motor cortex (M1), cingulate cortex, and other areas related to the integration of sensory information during gait imaginary.

Increasing evidence suggests that the neural mechanisms of executive function are primarily mediated by the frontal lobe, although the engagement of a particular subdivision seems to depend on the varied aspects of executive function (Stuss and Knight, 2013). For instance, the FPC has been theorized to enable the process of cognitive branching that allows the postponement of a pending task set for subsequent processes during the concurrent performance of the ongoing one (Koechlin and Hyafil, 2007). Given its domain-general function required for the simultaneous engagement of multiple tasks, we therefore chose to monitor the cerebral oxygenation in the FPC to examine the cognitive demands imposed by the examined DT walking conditions. In this study, the transient dynamic pattern illustrated in the time series of ΔHbO implied a reduction in neural engagement in the FPC. These observations are consistent with the recent work of Beurskens et al. (2014), where the elderly participants exhibited lower-than-baseline ΔHbO levels in the anterior and lateral PFC while performing a visually demanding secondary task on the treadmill. Decreased activation in the superior frontal gyrus, DLPFC, SMA, orbitofrontal gyrus, posterior cingulate cortex, and hippocampus in healthy individuals during treadmill walking has also been reported by Shimada et al. (2013) using positron emission tomography. Conversely, using a concurrent “talk” (alphabet recall) task, some studies found increased cerebral oxygenation in the PFC in healthy young adults (Holtzer et al., 2011) and elderly with mild cognitive impairment (Doi et al., 2013).

Although the underlying reasons for the observed reduction in PFC activation are difficult to determine, it could be at least partially explained by the neural plasticity and related to the cognitive network strategies utilized in dual-tasking (Reuter-Lorenz and Cappell, 2008; Barulli and Stern, 2013). According to the cascade-of-control model of executive function (Banich, 2009), the posterior DLPFC, the mid-DLPFC, and the posterior and anterior portions of the dorsal anterior cingulate cortex may be sequentially activated to maintain attentional sets to achieve the task goal. The theory further states that the extent of neural activation in the downstream brain regions is affected by the control effectiveness imposed by the upstream regions. In the literature, subdivisions of the PFC have been shown to be activated differently across participants while performing an *n*-back task (Hoshi et al., 2003). Individuals exhibiting non-sustained activation in the VLPFC were found to activate the VLPFC, DLPFC, and BA8 in a complementary manner. Moreover, the work of Lövdén et al. (2010) also implies that the brain might rely on the activation of compensatory networks instead of the primary cognitive network when confronted by challenging tasks. The concepts of neural compensation mechanisms were then adopted in theories used to explain differences in fMRI activation resulting from task difficulty or aging (Reuter-Lorenz and Cappell, 2008; Park and Reuter-Lorenz, 2009; Stern et al., 2012). Collectively, it is possible that different activation strategies between brain regions related to executive function may be utilized in response to a particular demand of cognitive resources in the examined scenario, thus leading to the hypoactivation observed in the right FPC. Unfortunately, due to the limitations of the fNIRS instrument used, cerebral oxygenation in other regions of PFC could not be determined in this study. Further research should seek to quantify the spatiotemporal neutral activities in those functionally connected areas for better understanding of multiple aspects of cognitive-motor interaction during DT walking.

## Limitations

As our participants were not required to hold the smartphone during NW as they did in DT walking, the observed changes in gait kinematics due to the effects of additional cognitive demands elicited by the *n*-back task cannot be separated from the ones imposed by smartphone holding. Reading texts from a smartphone, like texting, has been found to cause more synchronized motions between the head and the thorax segment but greater head movement relative to the global reference frame than unrestricted walking (Schabrun et al., 2014). These differences may disturb the dynamic balance and hence modify the joint coordination patterns of the lower extremities during ambulation. Moreover, the uncontrolled task prioritization among young adults could alter the potential DTC in either the cognitive or the gait task and reduce the statistical power of the study. However, recent studies have suggested that healthy young individuals tend to allocate most of their attention to the secondary task during DT walking unless they perceive undue challenges from the testing environments (Yogev-Seligmann et al., 2010; Schabrun et al., 2014; Plummer et al., 2015). Our data supported these findings, as the participants seemed to strive to maintain the *n*-back task accuracy by sacrificing the corresponding RT, along with reduced conscious control of gait characteristics. This tendency could be problematic when facing unexpected perturbation while walking in a real environment.

## Conclusion

The current findings provided the evidence that under DT walking the neural correlates of executive function and gait control tend to be modified in response to the cognitive resources imposed by road environment and the concurrent task. Healthy Young adults are inclined to focus on the challenging WM task and scarified gait performance to some extent through altered neural activations in the FPC and adapted coordination of lower-extremity kinematics. These distinguished changes across different levels of cognitive task complexity and walking road conditions demonstrated the nature of neuroplasticity and motor redundancy. The methodology described in this study to explore the DT effects and its underlying mechanisms may be extended to other activities commonly performed during walking in everyday life and lead to the development of novel interventions that could effectively improve the DT abilities.

## Author Contributions

M-IL conceived the study design, performed statistical analyses, interpreted the results, and drafted the manuscript. K-HL conducted the experiment, processed the data, and contributed to data interpretation. All authors read and approved the final manuscript.

## Conflict of Interest Statement

The authors declare that the research was conducted in the absence of any commercial or financial relationships that could be construed as a potential conflict of interest.

## ABBREVIATIONS

BA: Brodmann area
CV: coefficient of variation
DLPFC: dorsolateral prefrontal cortex
DT: dual task
DTC: dual-task costs
fMRI: functional magnetic resonance imaging
fNIRS: functional near-infrared spectroscopy
FPC: frontopolar cortex
HbO: oxygenated hemoglobin
HbR: deoxygenated hemoglobin
NW: normal walking
PFC: prefrontal cortex
rANOVA: repeated analysis of variance
RT: reaction time
SMA: supplementary motor area
VFT: verbal fluency task
VLPFC: ventrolateral prefrontal cortex
WM: working memory.
